# Phenolic Derivatives of *Artemisia Spicigera* C. Koch Growing in Iran

**Published:** 2015

**Authors:** Fariba Heshmati Afshar, Abbas Delazar, Hossein Nazemiyeh, Laleh Khodaie, Seddigheh Bamdad Moghaddam

**Affiliations:** a *Medical Philosophy and History Research Center, Tabriz University of Medical Sciences, Tabriz, Iran. *; b *Drug Applied Research Center, Tabriz University of Medical Sciences, Tabriz, Iran.*; c *School of Pharmacy, Tabriz University of Medical Sciences, Tabriz, Iran. *; d *Department of Traditional Pharmacy, Faculty of Traditional Medicine, Tabriz University of Medical Sciences, Tabriz, Iran. *

**Keywords:** *Artemisia spicigera*, phenolic compounds, Phytochemistry, Asteraceae

## Abstract

This study aimed to determine phenolic compounds of *Artemisia spicigera* (family Asteraceae) growing in East-Azarbaijan province of Iran. 20%, 40 % and 60% SPE fractions of methanolic extract of *A. spicigera*, were subjected to reversed phase preparative HPLC, with the mobile phase consisted of methanol and water. Structural identification of phytochemicals by spectroscopic methods including UV and NMR spectroscopy, yielded 4, 6-di methoxy acetophenone-2-O-β-D-glucopyranoside from 20%, 5-methoxyluteolin 7-*O*-β-D-glucopyranoside, luteolin and chrysoeriol 7-O-β-D-glucopyranoside from 40% and 5-methoxy luteolin from 60% SPE fractions. Although within identified pure compounds, luteolin was the only phenolic reported from some other species of *Artemisia*, but occurrence of remained identified phenolics in this study, was firstly reported from *Artemisia* genus. Further phytochemical investigations were proposed in order to isolate some other active fractions and pure compounds.

## Introduction

*Artemisia* genus (Asteraceae) with common Persian name of "Dermaneh" includes 200-400 species in the world that 34 species of it belong to Iran (1). Plants of this genus have been widely used in Iranian Traditional Medicine (ITM) as carminative, diuretic, anthelmintic and also as a cure for insect bites, difficulty in breathing, cramps and fever (2). Modern pharmacological researches have been indicated antimalarial (3-5), antibacterial (6), antifungal (7-8), antiviral (9-10), antioxidant (11), anti-inflammatory (12-14), antipyretic (14), analgesic (12), antiproliferative (15-16), cytotoxic (17), anticancer (18) activities by various species of *Artemisia* genus. Various phytochemical constituents such as sesquiterpenoids, flavonoids, coumarins, triterpenoids, steroids, purines, phenolics, lipids and aliphatic compounds and monoterpenoids (19-22) were isolated from different species of this genus. *Artemisia spicigera* known as ''dermane ye sonbolei'' in Persian language, is an aromatic herb growing in Northern and North-western parts of Iran (1). According to previous studies antioxidant, total phenol and flavonoid contents of this plant have been reported (23). In fact, the results of mentioned article indicated that 40% and 60% methanol in water fractions demonstrated maximum antioxidant, total phenol and flavonoids content. So far, however there has been no report on phenolic compounds of this plant, thus the purpose of this paper is to focus on identification of phenolics from methanolic extract of *A. spicigera*.

## Experimental

*Plant material*

Arial parts of *Artemisia spicigera* C. Koch were collected from Julfa and border of Aras River in East Azarbaijan province, Iran in 2009. A Voucher specimen for this collection (TBZ-FPH 716) has been deposited at the Herbarium of faculty of pharmacy, Tabriz University of Medical Sciences, Tabriz, Iran.

*Extraction*

Air dried and powdered arial parts of *A. spicigera *(100 g) were successively soxhelet extracted using *n*-hexan (8 h), dichloromethan (DCM) (10 h) and methanol (MeOH) (8 h). Obtained extracts were separately concentrated under vaccum by rotary evaporator at an ambient temperature yielding 6.41 g, 1.45 g and 8.63 g from each extract respectively.

*Fractionation of methanolic extract*

A portion of dried methanolic extract (2 g) was fractionated by solid-phase-extraction (SPE) on Sep-Pak (Vac 35 cc; 10 g; C18 cartridge) using a step gradient of MeOH-water mixture (10: 90, 20: 80, 40: 60, 60: 40, 80: 20 and 100: 0), 200 ml each. Solvents of eluted fractions were removed using a rotary evaporator at a temperature not exceeding 45ºC. In order to increase the available SPE fractions, procedure was repeated with extra 2 g of methanolic extract yielding 1570, 444, 794, 422, 65 and 670 mg of each fraction, respectively.

*Isolation and identification of compounds*

**Figure 1:**
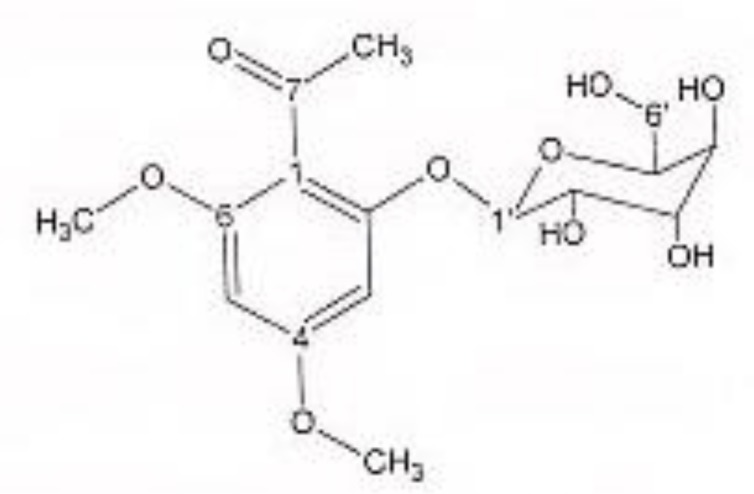
4, 6-di methoxy acetophenone-2-O-β-D-glucopyranoside.

**Figure 2:**
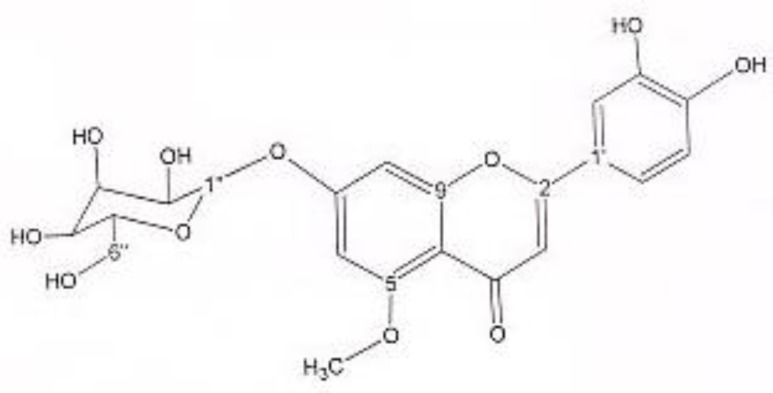
5-methoxyluteolin 7-O-β-D-glucopyranoside.

**Figure 3:**
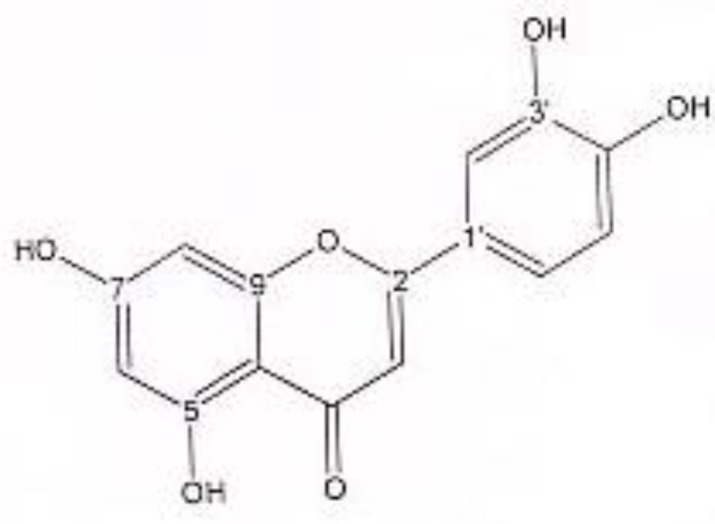
luteolin.

**Figure 4:**
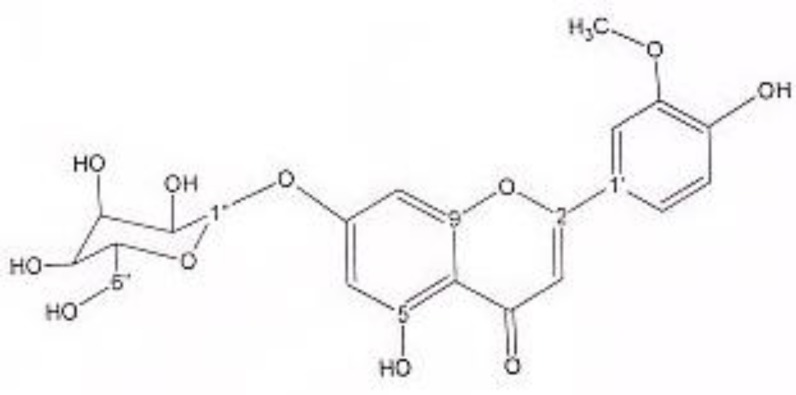
chrysoeriol 7-O- β-D-glucopyranoside.

**Figure 5:**
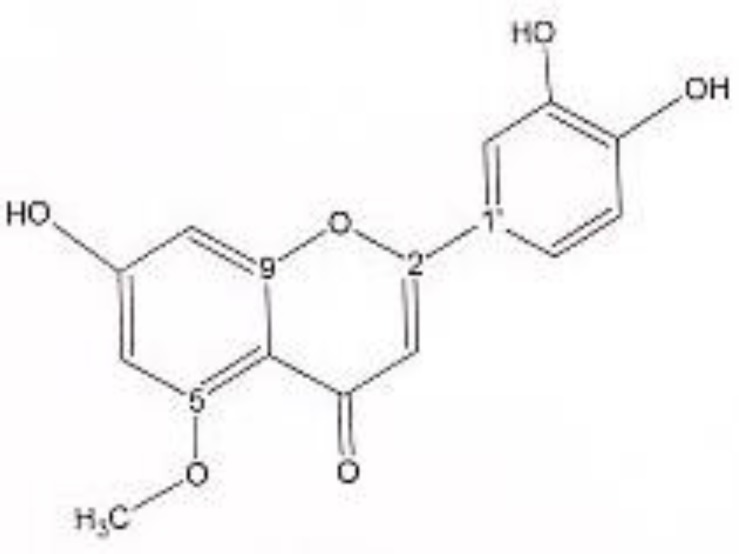
5-methoxy luteolin.

In order to isolate phytochemicals of 20%, 40% and 60% MeOH in water fractions they were subjected to reversed phase preparative HPLC (Knauer, preparative pump 1800),with photodiode array detector (PDA), equipped with a Reprosil 100 C18 (250 mm length, 20 mm i.d, particle size 10 µm, Dr. Maisch, Germany) column. The mobile phase consisted of (A) methanol and (B) water. For isolation of 10.3 mg of 4, 6-di methoxy acetophenone-2-O-β-D- glucopyranoside (Figure 1) from 20% SPE fraction, following mobile phase program was used over 63 min: 15% A was initially changed to 40% within 50 min and maintained there for 13 min. A program over a run time of 63 min was applied for separation of 5.1 mg of 5-methoxyluteolin 7-*O*-β-D-glucopyranoside (Figure 2), 5 mg of luteolin (Figure 3) and 5 mg of chrysoeriol 7-O-β-D-glucopyranoside (Figure 4) from 40% SPE fraction: 30% A was changed to 80% within 50 min and it stayed there for 13 min. A program over a run time of 63 min was applied for separation of 9.6 mg of 5-methoxy luteolin (Figure 5) from 60% fraction: 45% A was changed to 75% within 30 min and it maintained there for 33 min. The flow rate was 8 ml/min and the injection volume was 1 ml. Structures of compounds were elucidated by ^1^H and ^13^C NMR (Bruker-spectrospin at 200 MHz) as well as comparison with the literature data of respective compounds (24-29). Furthermore Ultra Violet spectral analysis of flavonoids by UV spectrophotometer instrument (Shimadzu 1800), as well as shift reagents confirmed structures of identified ones by establishing the site of attachments (30). Shift reagents were as follows: Sodium Methoxide (NaOMe) (Merck), Aluminum Chloride (AlCl_3_), Aluminum Chloride/Chloridric acid (AlCl_3_/HCl) (Merck) Sodium Acetate (NaOAC) and Sodium Acetate/Boric acid (NaOAC/H_3_BO_3_) (Merck).

^1^HNMR and ^13^CNMR of 4, 6-di methoxy acetophenone-2-O-β-D-glucoside was as follows (Figure. 1): HNMR (200 MHz, CD3OD): Aglycone moiety: δ 6.50 (1H, d, J = 2.03 Hz, H3), δ 6.32 (1H, d, J = 2.03 Hz, H5), δ 3.81 (3H, S, 9-OMe), δ 3.79 (3H, S, 10-OMe), δ 2.49 (3H, S, H8). Glucose moiety: δ 4.37 (lH, d, J = 7.8Hz, Hl'), 3.2-3.8 (signal patterns unclear due to over lapping, H2'-3'-4'-5'-6'). ^13^CNMR (200 MHz, CD3OD). Aglycone moiety: δ 198.01 (C7), 162.07 (C2), 161.25 (C6), 159.33 (C4), 113.04 (C1), δ 94.03 (C5), δ 92.67 (C3), δ 54.94 (C9-OMe), δ 54.66 (C10-OMe), δ 31.50 (C8). Glucose moiety: δ 102.50 (C1'), δ 77.08 (C3'), δ 76.59 (C5'), δ 73.48 (C2'), δ 69.95 (C4'), δ 62.5 (C6').

^1^HNMR and ^13^CNMR of 5-methoxyluteolin 7-*O*-β-D-glucopyranoside was as follows (Figure 2): HNMR (200 MHz, CD3OD): Aglycone moiety: δ 7.58 (1H, dd, J = 8.20, 2 Hz, H6'), δ 7.52 (1H, d, J = 2 Hz, H2'), δ 6.96 (1H, d, J = 8.20 Hz, H5'), δ6.85 (1H, d, J = 2 Hz, H8), δ 6.73 (1H, S, H3), δ 6.50 (1H, d, J = 2 Hz, H6), δ 3.97 (3H, S, O-Me). Glucose moiety: δ 4.39 (lH, d, J = 7.8Hz, Hl''), 3.2-3.9 (signal patterns unclear due to over lapping, H2''-3''-4''-5''-6''). ^13^CNMR (200 MHz, CD3OD). Aglycone moiety: δ 182.39 (C4), δ 164.85 (C7), δ 164.62 (C2), δ 161.77 (C5), δ 157.93 (C9), δ 149.55 (C4'), δ 145.59 (C3'), δ 122.20 (C1'), δ 118.88 (C6'), δ 115.37 (C5'), δ 112.74 (C2'), δ 102.93 (C10), δ 102.43(C3), δ 98.71 (C6), δ 93.59 (C8), δ 56.96 (C5-OMe). Glucose moiety: δ 98.15 (C1''), δ 76.98 (C5''), δ 76.54 (C3''), δ 72.45 (C2''), δ 70.93 (C4''), δ 62.99 (C6''). UV spectrum, bands ІІ and І respectively (MeOH, λ_max_, nm): 273, 330; + NaOMe 262, 382; + AlCl_3_ 267, 355; + AlCl_3_/HCl 273, 330; + NaOAC 271, 375; + NaOAC/H_3_BO_3_ 265, 355. UV spectrum of compound 2 with MeOH as solvent is characteristic of flavone derivatives. Studying UV spectra data after addition of NaOMe and production of 52 nm band І bathochromic shift, is indicative of 4'-OH. Addition of AlCl_3_ produced 25 nm band І bathochromic shift. After addition of HCl band І returned to previous model. Thus ortho di-hydroxyl structure of compound can be elucidated. Addition of NaOAC did not produce any band ІІ bathochromic shift which was consistant with glycosylation of 7-OH.

^1^HNMR and ^13^CNMR of luteolin was as follows (Figure 3): Aglycone moiety: δ 7.58 (1H, dd, J = 7.95, 2 Hz, H6'), δ 7.50 (1H, d, J = 2 Hz, H2'), δ 6.96 (1H, d, J = 7.95 Hz, H5'), δ6.46 (1H, d, J = 2 Hz, H8), δ 6.63 (1H, S, H3), δ 6.21 (1H, d, J = 2 Hz, H6). Aglycone moiety: δ 182.49 (C4), δ 164.85 (C7), δ 164.63 (C2), δ 161.70 (C5), δ 157.93 (C9), δ 149.55 (C4'), δ 145.59 (C3'), δ 122.20 (C1'), δ 118.85 (C6'), δ 115.32 (C5'), δ 112.69 (C2'), δ 103.00 (C10), δ 102.37 (C3), δ 97.81 (C6), δ 93.50 (C8). UV spectrum, bands ІІ and І respectively (MeOH, λ_max_, nm): 268, 340; + NaOMe 267, 402; + AlCl_3_ 274, 418; + AlCl_3_/ HCl 278, 385; + NaOAC 273, 396; + NaOAC/H_3_BO_3_ 267, 360. UV spectrum of compound 2 with MeOH as solvent is characteristic of flavone derivatives. Studying UV spectra data after addition of NaOMe and production of 62 nm band І bathochromic shift was consistant with 4'-OH. Addition of AlCl_3_ produced 78 nm band І bathochromic shift. Addition of HCl, caused band І to return to previous model, indicative of ortho di-hydroxyl structure of compound. Addition of NaOAC and production of band ІІ bathochromic shift of 5 nm indicated free 7-OH position of flavonoid (no glycosylation). 

^1^HNMR and ^13^CNMR of chrysoeriol 7-O-beta-D-glucoside was as follows (Figure 4): HNMR (200 MHz, CD3OD): Aglycone moiety: δ 7.52 (1H, dd, J = 7.95Hz, 2 Hz, H6'), δ 7.50 (1H, S, H2'), δ 6.96 (1H, d, J = 7.95 Hz, H5'), δ 6.73 (1H, S, H3), δ 6.46 (1H, d, J = 2 Hz, H8), δ 6.21 (1H, d, J = 2 Hz, H6). Glucose moiety: δ 4.41 (lH, d, J =7.8Hz, Hl''), 3.2-3.9 (signal patterns unclear due to over lapping, H2''-3''-4''-5''-6''). ^13^CNMR (200 MHz, CD3OD). Aglycone moiety: δ 181.65 (C4), δ 164.42 (C2), δ 160.7 (C5), δ 163.59 (C7), δ 159.93 (C9), δ 148.55 (C4'), δ 146.52 (C3'), δ 123.26 (C1'), δ 117.88 (C6'), δ 115.67 (C5'), δ 111.74 (C2'), δ 103.43 (C10), δ 102.53(C3), δ 96.90 (C6), δ 95.59 (C8), δ 56.01 (O-Me). Glucose moiety: δ 98.15 (C1''), δ 77.00 (C5''), δ 76.44 (C3''), δ 72.65 (C2''), δ 70.86 (C4''), δ 63.01 (C6''). UV spectrum bands ІІ and І respectively (MeOH, λ_max_, nm): 277, 340; + NaOMe 282, 400; + AlCl_3_ 274, 355; + AlCl_3_/HCl 273, 353; + NaOAC 277; + NaOAC/H_3_BO_3_ 277, 340. UV spectrum of compound 2 with MeOH as solvent is characteristic of flavones derivatives. Studying UV spectra data after addition of NaOMe and production of 60 nm band І bathochromic shift, is indicative of 4'-OH. After addition of AlCl_3_ and production of 15 nm band І bathochromic shift and no change in absorption maxima after addition of HCl, exhibited that this compound did not consist of ortho di-hydroxyl structure. Addition of NaOAC did not produce any band ІІ bathochromic shift indicating glycosylation of 7-OH position.

^1^HNMR and ^13^CNMR of 5-methoxy luteolin was as follows (Figure 5): Aglycone moiety: δ 7.39 (1H, dd, J = 8.30, 2 Hz, H6'), δ 7.37 (1H, d, J = 2 Hz, H2'), δ 6.96 (1H, d, J = 8.30 Hz, H5'), δ6.51 (1H, S, H3), δ6.39 (1H, d, J = 2 Hz, H8), δ 6.16 (1H, d, J = 2 Hz, H6), δ 3.89 (O-Me). Aglycone moiety: δ 182.49 (C4), δ 164.33 (C2), δ 163.85 (C7), δ 159.93 (C9), δ 159.70 (C5), δ 147.45 (C4'), δ 144.50 (C3'), δ 123.50 (C1'), δ 117.85 (C6'), δ 116.32 (C5'), δ 113.68 (C2'), δ 105.00 (C10), δ 103.37 (C3), δ 95.81 (C6), δ 94.50 (C8), δ 56.23 (O-Me). UV spectrum: bands ІІ and І respectively (MeOH, λ_max_, nm): 268, 330; + NaOMe 276, 387; + AlCl_3_ 274, 414; + AlCl_3_/HCl 279, 333; + NaOAC 273, 400; + NaOAC/H_3_BO_3_ 267. UV spectrum of compound 2 with MeOH as solvent is characteristic of flavones derivatives. Studying UV spectra data after addition of NaOMe and production of band І bathochromic shift of 57 nm, is consistant with 4'-OH. Addition of AlCl_3_ caused 84 nm bathochromic shift. After addition of HCl, band І returned to previous model, indicative of ortho di-hydroxyl structure of compound. Addition of NaOAC and production of band ІІ bathochromic shift of 5 nm was consistent with 7-OH.

## Results and Discussion

Solid phase extraction (SPE) followed by reversed-phase prep-HPLC analysis of the arial parts of the SPE fractions (20%, 40% and 60%) yielded one acetophenone as well as four flavonoids, which were 5-methoxyluteolin 7-O-β-D-glucopyranoside, luteolin, chrysoeriol 7-O-β-D-glucoside and 5-methoxy luteolin. Structural identification of isolated phytochemicals was carried out by UV and NMR analysis and all spectroscopic data were in agreement with respective published data. According to our knowledge, none of these phenolic compounds were isolated from *A. spicigera.* Although in prior studies sesquiterpene lactones have been reported from this plant (31), this is the first report on the isolation and identification of phenolic compounds from *A. spicigera. *In previous studies, antioxidant activity by DPPH assay, as well as total phenol and total flavonoid content of the MeOH extract of *A. spicigera* and its solid phase extracted fractions, has been reported. Results of this research showed that proportion of flavonoids and total phenols of 40% and 60% SPE fractions was higher, compared to other fractions and the mentioned active fractions indicated better antioxidant activity as well. Since good radical scavenging activity is associated with different classes of plant phenolics (32-36) so findings of this study is parallel with results of previous works. It means that isolation and identification of phenolics which are one acetophenone and four flavonoids from 20%, 40% and 60% SPE fractions justified higher antioxidant activity as well as higher total phenol and flavonoid content of these fractions.

Acetophenone derivatives are highly distributed in different species of *Artemisia* genus (37-42), but to our knowledge, 4, 6-dimethoxyacetophenone-2-*O*-β--glucopyranoside was isolated and identified from *Artemisia* genus for the first time. The second compound, 5-methoxyluteolin 7-O-β-D-glucopyranoside, was previously isolated from *Urtica dioica* Maxim. (25), while this was the first report on isolation of this flavonoid from *Artemisia* genus. Within flavonoids Not only 5-methoxyluteolin 7-O-β-D-glucopyranoside but also chrysoeriol 7-glucoside has not been reported from this genus. Instead, within the genus *Artemisia* distribution of flavonoids, especially luteolin appears to be widespread (42-45).

## Conclusion

It can be concluded that pharmacodynamics studies should be undertaken to establish the mechanism of action of isolated compounds for understanding the reason of their usage in traditional and folk medicine. Hence, further phytochemical investigations will be proposed in order to isolate some other active fractions and pure compounds.

## References

[1] Mozaffarian V (1386). A Dictionary of Iranian Plant Names.

[2] Khorasani MA (2001). Makhzan al Advieh. Choogan Press.

[3] Liu KCSC, Yang SL, Roberts M, Elford B, Phillipson J (1992). Antimalarial activity of Artemisia annua flavonoids from whole plants and cell cultures. Plant Cell Rep..

[4] Mojarrab M, Naderi R, Afshar FH (2015). Screening of Different Extracts from Artemisia Species for Their Potential Antimalarial Activity. Iran J. Pharm. Res..

[5] Abdin M, Israr M, Rehman R, Jain S (2003). Artemisinin, a novel antimalarial drug: biochemical and molecular approaches for enhanced production. Planta Med..

[6] Habibi Z, Yousefi M, Mohammadi M, Eftekhar F, Biniyaz T (2010). Chemical composition and antibacterial activity of the volatile oils from Artemisia turcomanica. Chem. Nat. Compd..

[7] Kordali S, Kotan R, Mavi A, Cakir, Ala A (2005). Determination of the chemical composition and antioxidant activity of the essential oil of Artemisia dracunculus and of the antifungal and antibacterial activities of Turkish Artemisia absinthium, A. dracunculus, Artemisia santonicum, and Artemisia spicigera essential oils. J. Agric. Food Chem..

[8] Meepagala KM, Sturtz G, Wedge DE (2002). Antifungal constituents of the essential oil fraction of Artemisia dracunculus L. var. dracunculus. J. Agric. Food Chem..

[9] Sinico C, DeLogu A, Lai F, Valenti D, Manconi M (2005). Liposomal incorporation of Artemisia arborescens L. essential oil and in-vitro antiviral activity. Eur. J. Pharm. Biopharm..

[10] Abid AKM, Jain D, Bhakuni R, Zaim M, Thakur R (1991). Occurrence of some antiviral sterols in Artemisia annua. Plant Sci..

[11] Juteau F, Masotti V, Bessiere JM, Dherbomez M, Viano J (2002). Antibacterial and antioxidant activities of Artemisia annua essential oil. Fitoterapia.

[12] Moscatelli V, Hnatyszyn O, Acevedo C, Megías J, Alcaraz MJ (2006). Flavonoids from Artemisia copa with anti-inflammatory activity. Planta Med..

[13] Ahmad F, Khan RA, Rasheed S (1992). Study of analgesic and anti-inflammatory activity from plant extracts of Lactuca scariola and Artemisia absinthium. J. Islam. Acad. Sci..

[14] Moran A, Martin M, Montero M, de Urbina A, Sevilla M (1989). Analgesic, antipyretic and anti-inflammatory activity of the essential oil of Artemisiacaerulescens subsp. gallica. J. Ethnopharmacol..

[15] Taghizadeh Rabe SZ, Mahmoudi M, Ahi A, Emami SA (2011). Antiproliferative effects of extracts from Iranian Artemisia species on cancer cell lines. Pharm. Biol..

[16] Tilaoui M, Mouse HA, Jaafari A, Aboufatima R, Chait A (2011). Chemical composition and antiproliferative activity of essential oil from aerial parts of a medicinal herb Artemisia herba-alba. Rev. Bras. Farmacogn..

[17] Foray L, Bertrand C, Pinguet F, Soulier M, Astre C (1999). In-vitro cytotoxic activity of three essential oils from Salvia species. J. Essent. Oil Res..

[18] Emami SA, Vahdati MN, Oghazian M, Vosough R (2009). The anticancer activity of five species of Artemisia on Hep2 and HepG2 cell lines. Pharmacol. Online.

[19] Nibret E, Wink M (2010). Volatile components of four Ethiopian Artemisia species extracts and their in-vitro antitrypanosomal and cytotoxic activities. Phytomedicine..

[20] Brisibe EA, Umoren UE, Brisibe F, Magalhäes PM, Ferreira JF (2009). Nutritional characterisation and antioxidant capacity of different tissues of Artemisia annua L. Food chem..

[21] Sadiq A, Hayat MQ, Ashraf M (2014). Ethnopharmacology of Artemisia annua L.: A Review.

[22] Gholamrezaie LS, Mohammadi M, Jalali Sendi J, Abolghasemi S, Roostaie AMM (2013). Extract and leaf powder effect of Artemisia annua on performance, cellular and humoral immunity in broilers. Iran J. Vet. Res..

[23] Afshar FH, Delazar A, Nazemiyeh H, Esnaashari S, Moghadam SB (2012). Comparison of the total phenol, flavonoid contents and antioxidant activity of methanolic extracts of Artemisia spicigera and A. splendens growing in Iran. Pharm. Sci..

[24] Ghosal S, Mittal P, Kumar Y, Singh SK (1989). Free and glucosyloxy acetophenones from Pancratium biflorum. Phytochem..

[25] Zhou Y, Wang W, Tang L, Yan XG, Shi LY (2009). Lignan and flavonoid glycosides from Urtica laetevirens Maxim. J. Nat. Med..

[26] Mabry TJ, Markham KR, Thomas MB (1970). The Systematic Identification of Flavonoids.

[27] Ferreira JF, Luthria DL, Sasaki T, Heyerick A (2010). Flavonoids from Artemisiaannua L. as antioxidants and their potential synergism with artemisinin against malaria and cancer. Molecules.

[28] Hartwig UA, Maxwell CA, Joseph CM, Phillips DA (1990). Chrysoeriol and luteolin released from alfalfa seeds induce nod genes in Rhizobium meliloti. Plant Physiol..

[29] Chi F, Deng J, Wang Y (2010). Chemical constituents from Lagotis brevituba.

[30] Andersen OM, Markham KR (2010). Flavonoids: Chemistry, Biochemistry and Applications.

[31] Marco JA, Sanz JF, Sancenon F, Rustaiyan A, Saberi M (1993). Sesquiterpene lactones from Artemisia species. Phytochem..

[32] Cai YZ, Sun M, Xing J, Luo Q, Corke H (2006). Structure–radical scavenging activity relationships of phenolic compounds from traditional Chinese medicinal plants. Life Sci..

[33] Miliauskas G, Venskutonis P, Van TB (2004). Screening of radical scavenging activity of some medicinal and aromatic plant extracts. Food Chem..

[34] Madani MSN, Delazar A, Nazemiyeh H, Khodaie L (2015). Biological Activity and Phytochemical Study of Scutellaria platystegia. Iran J. Pharm. Res..

[35] Meda A, Lamien CE, Romito M, Millogo J, Nacoulma OG (2005). Determination of the total phenolic, flavonoid and proline contents in Burkina Fasan honey, as well as their radical scavenging activity. Food Chem..

[36] Zhao DB, Li LX, Liu XH, Li MJ, Wang WL (2007). Two new phenolic compounds from Artemisia sphaerocephala. Chinese Chem. Lett..

[37] Singh AK, Pathak V, Agrawal PK (1997). Annphenone, a phenolic acetophenone from Artemisia annua. Phytochem..

[38] Rauter AP, Branco I, Tosrão Z, Pais MS, Gonzalez AG (1989). Flavonoids from Artemisia campestris subsp. maritima.. Phytochem..

[39] De JPT, Gonzalez M, Muriel M, Bellido I (1984). Phenolic derivatives from Artemisia campestris subsp. glutinosa. Phytochem..

[40] Gonzalez A, Bermejo J, Estevez F, Velazquez R (1983). Phenolic derivatives from Artemisia glutinosa. Phytochem..

[41] Hammouda F, Rizk A, Ismail S, Hassan N (2013). Isolation of an acetophenone derivative and coumarins from Artemisia monosperma. Del. Fitoterapia.

[42] Brown D, Asplund RO, McMahon VA (1975). Phenolic constituents of Artemesiatridentate spp. vaseyana. Phytochem..

[43] Lee SJ, Chung HY, Maier CGA, Wood AR, Dixon RA (1998). Estrogenic flavonoids from Artemisia vulgaris L. J. Agric. Food Chem..

[44] Liu YL, Mabry T (1981). Flavonoids from Artemisia frigid. Phytochem.

[45] Yang SL, Roberts MF, O'Neill MJ, Bucar F, Phillipson JD (1995). Flavonoids and chromenes from Artemisia annua. Phytochem..

